# Supplementary material for: Neural Patterns of Social Pain in the Brain‐Wide Representations Across Social Contexts
Source: Adv Sci (Weinh). 2025 Mar 17;12(18):2413795. doi: 10.1002/advs.202413795 (PMC12079339; doi:10.1002/advs.202413795)
Supplement: Supplementary file 1 — Supporting Information [file ADVS-12-2413795-s001.docx]

Neural patterns of social pain in the brain-wide representations across social contexts

Xiaodong Zhang, Peng Qing, Qi Liu, Can Liu, Lei Liu, Xianyang Gan, Kun Fu, Chunmei Lan, Xinqi Zhou, Keith M. Kendrick, Benjamin Becker, Weihua Zhao*

XD. Zhang, P. Qing, Q. Liu, C. Liu, L. Liu, X. Gan, K. Fu, C. Lan, K.M.K., W. Zhao

The Center of Psychosomatic Medicine, Sichuan Provincial Center for Mental Health, Sichuan Provincial People’s Hospital

University of Electronic Science and Technology of China

Chengdu, 611731, China

E-mail: [zarazhao@uestc.edu.cn](mailto:zarazhao@uestc.edu.cn)

XQ. Zhou

Institute of Brain and Psychological Sciences

Sichuan Normal University

Chengdu, 610066, China

B.B.

Department of Psychology, State Key Laboratory of Brain and Cognitive Sciences

The University of Hong Kong

Hong Kong, 999077, China.

This file includes:

Supporting Text

Figures S1 to S6

Tables S1 to S6

# Supporting results

## 1.1 Behavioral results

Both social exclusion (Ex, M = 2.89, SD = 0.75) and separation (Sep, M = 3.48, SD = 0.73) videos elicited significant negative empathic responses based on self-reported pain ratings, compared to their respective positive (Inclusion-Inc: M = 1.26, SD = 0.33; Company-Com: M = 1.20, SD = 0.30; Ex vs. Inc: *t_(64)_* = 18.674, *p_FDR_* < 0.001, Cohen’s *d* = 2.82; Sep vs. Com: *t_(64)_* = 25.226, *p_FDR_* < 0.001, Cohen’s *d* = 4.07) and neutral conditions (Ex_control: M = 1.44, SD = 0.46; Sep_control: M = 1.33, SD = 0.41; Ex vs. Ex_control: *t_(64)_* = 19.124, *p_FDR_* < 0.001, Cohen’s *d* = 2.33; Sep vs. Sep_control: *t_(64)_* = 27.151, *p_FDR_* < 0.001, Cohen’s *d* = 3.63). However, the pain empathy response induced by the separation was stronger than that induced by the exclusion videos (*t_(64)_* = 9.47, *p_FDR_* < 0.001, Cohen’s *d* = 0.80, **Figure 2A**). Additionally, there was a significant difference between exclusion and separation video clips in terms of arousal ratings (*t_(64)_* = 5.399, *p_FDR_*< 0.001) but not intensity ratings (*t_(64)_* = 1.597, *p _FDR_* = 0.33).

# Supporting figures

**
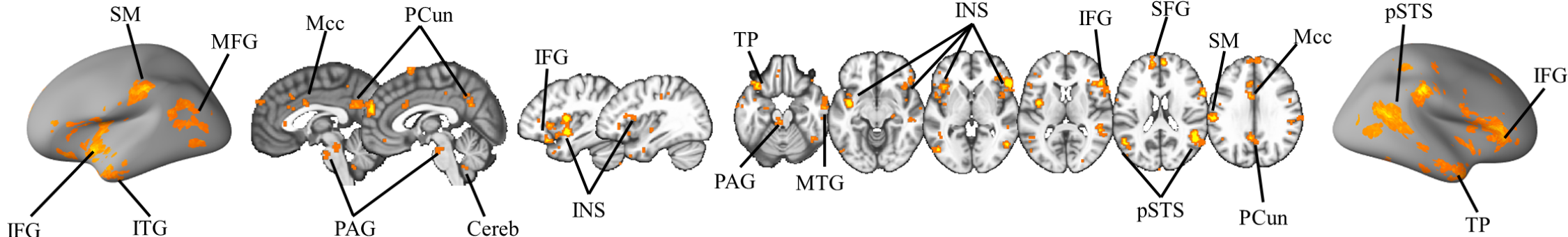
**

**Supporting Figure S1. ROIs of Neurosynth.** We utilized term-based meta-analyses from Neurosynth to generate a whole-brain region of interest focused on “Empathy”. This included IFG, ITG, SM, MFG, Mcc, PAG, PCun, Cereb, INS, MTG, SFG, pSTS, TP and other brain regions. IFG, Inferior Frontal Gyrus; ITG, Inferior Temporal Gyrus; SM, Supramarginal Gyrus; MFG, Middle Frontal Gyrus; Mcc, Middle Cingulate Cortex; PAG, Periaqueductal Gray; PCun, Precuneus; Cereb, Cerebellum; INS, Insula; MTG, Middle Temporal Gyrus; SFG, Superior Frontal Gyrus; pSTS, posterior Superior Temporal Sulcus; TP, Temporal Pole.


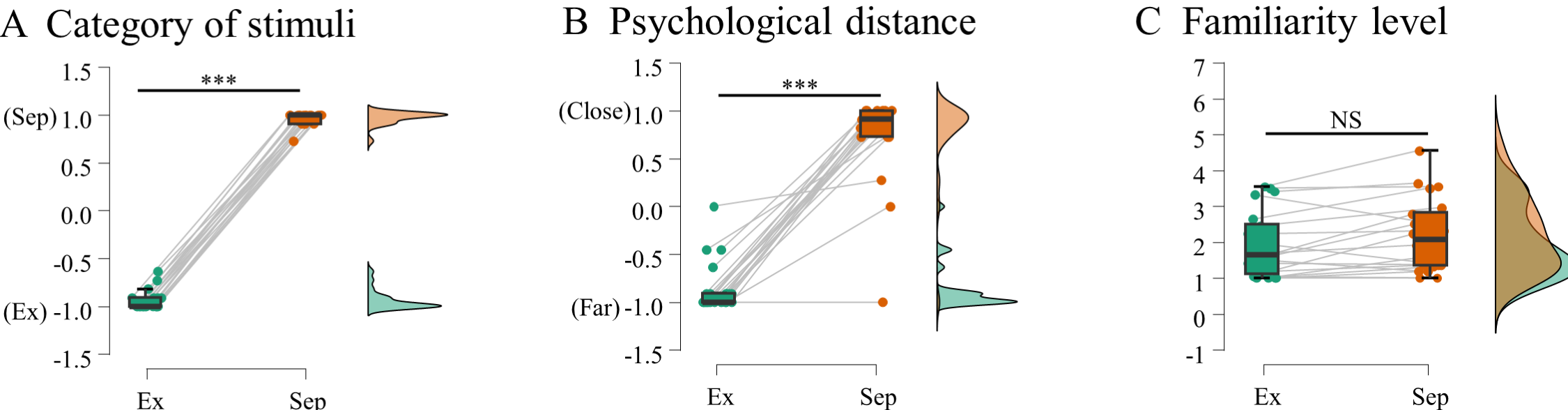


**Supporting Figure S2. Behavioral ratings for the stimuli using an independent sample of 21 participants. (A)** All participants were able to accurately identify the stimuli types for in total of 44 video clips (22 social exclusion stimuli, 22 social separation stimuli) **(B)** Regarding the psychological distance, 20 (95%) participants perceived the psychological distance between characters in the video clips depicting social exclusion as distinct, and 18 (86%) participants perceived the psychological distance of social separation stimulus as close. **(C)** Familiarity ratings (1-7 points) for the exclusion and separation stimuli. ***P<0.001, all p values are FDR-corrected. NS, no significant.


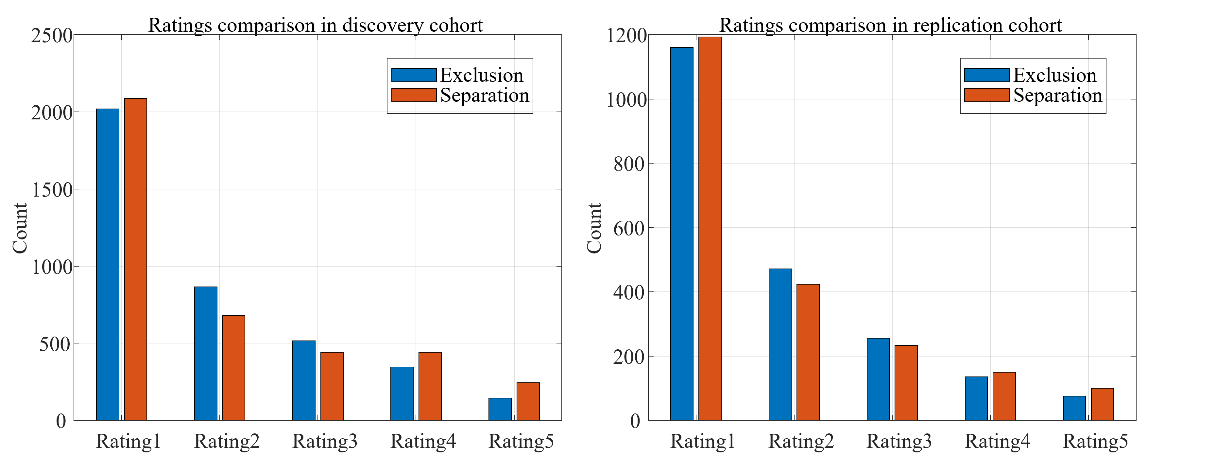


**Supporting Figure S3. Self-reported empathy levels.** The graph on the left shows the total number of ratings for each level of the discovery cohort (n = 65); the graph on the right shows the result of the replication cohort (n = 35).

**
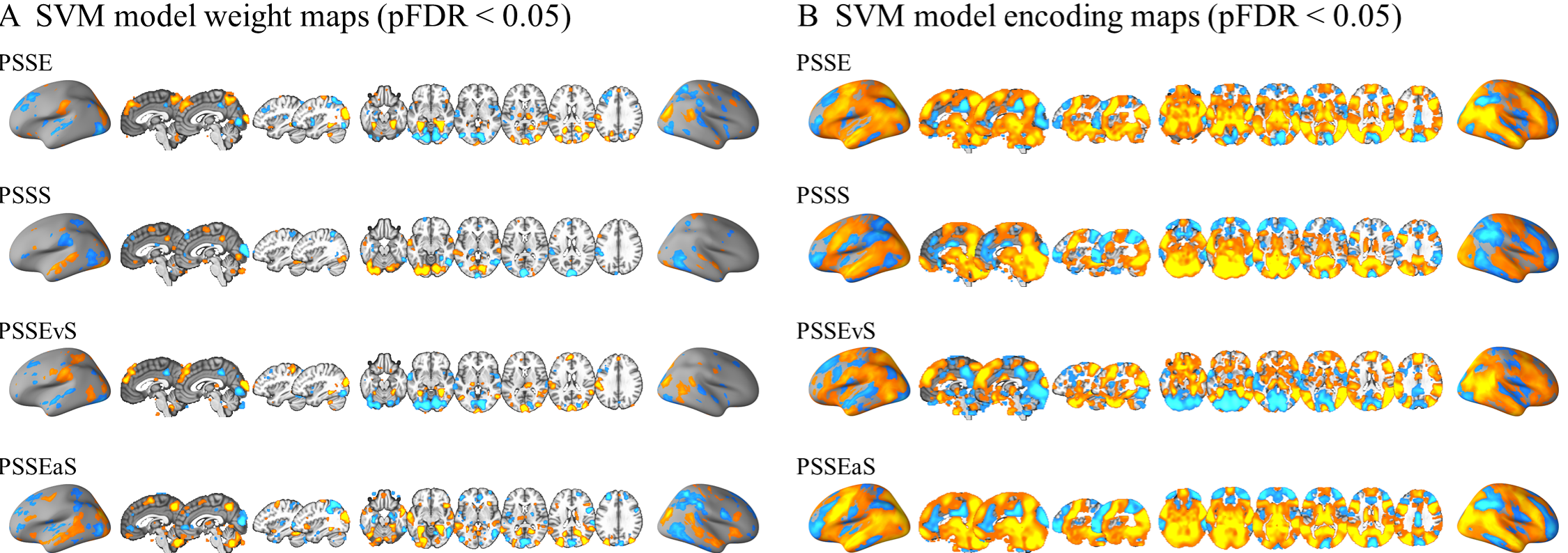
**

**Supporting Figure S4. Model weight maps and model encoding maps for each SVM pattern. (A)** The maps show the model weights to indicate the brain regions that consistently contribute to the classification of social pain empathy (based on bootstrap tests with 10,000 iterations, shown here at *p_FDR_* < 0.05). **(B)** The model encoding map shows the brain regions whose voxel activity correlates with SVM model outcomes (*p_FDR_* < 0.05).

**
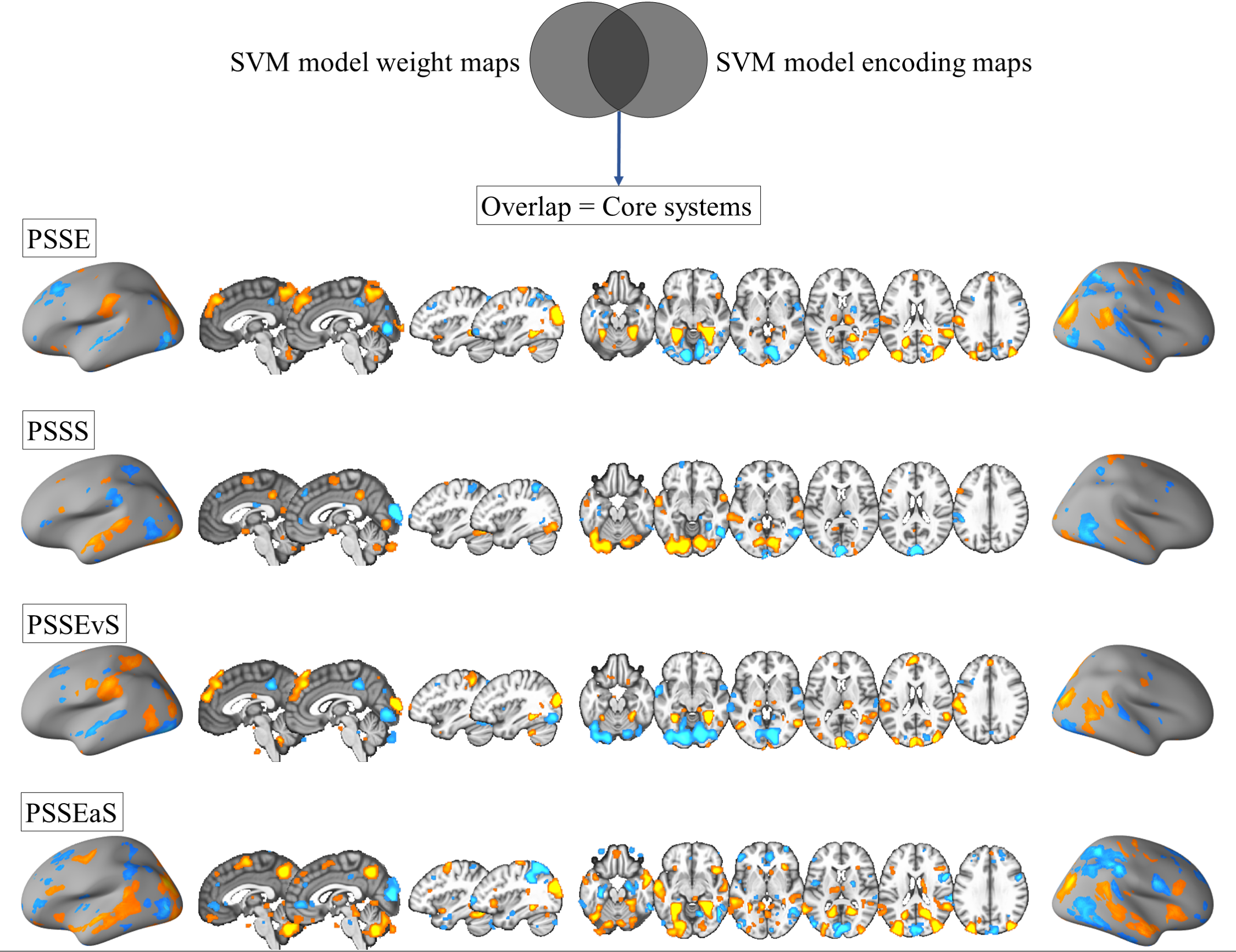
**

**Supporting Figure S5. Core systems involved in social pain empathy.** The map shows the core system of each model by overlapping the SVM pattern weight map (**Figure S4A**) with the encoding map of that model (**Figure S4B**). The final graph threshold is set at k > 10 (preserving clusters with voxel values greater than or equal to 10).

**
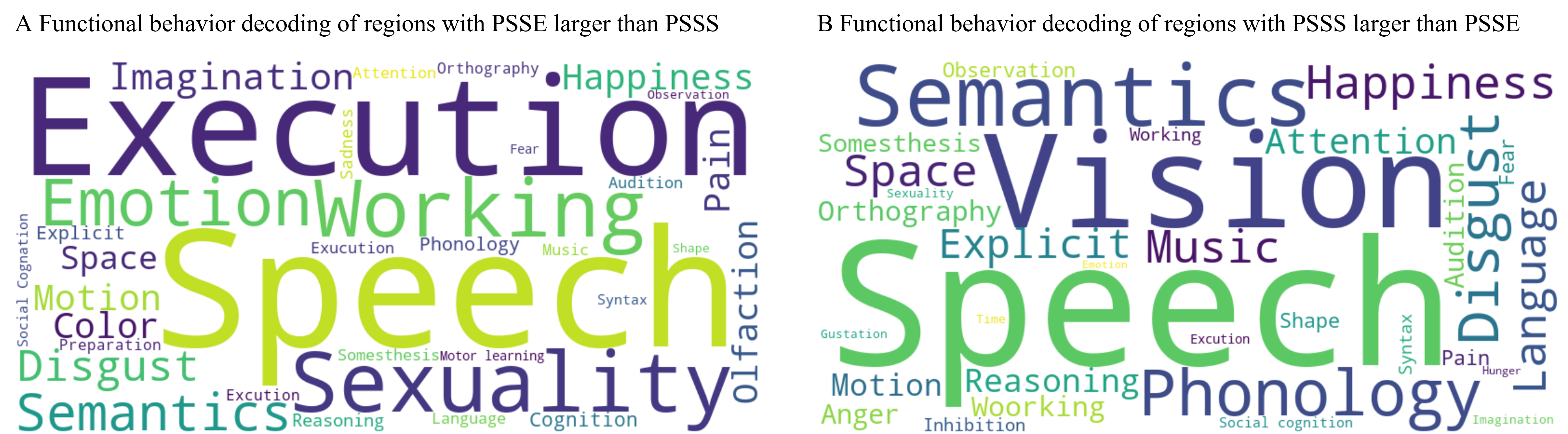
**

**Supporting Figure S6. Functional behavior decoding.** (A-B) Functional behavior decoding of brain regions that more predictive of pain empathy evoked by social exclusion (A) and social separation (B).

# Supporting Tables

**Supporting Table S1.** **Intensity rating, arousal rating, and pain rating.**

| Metrics | Type of stimulus | P-value |
| --- | --- | --- |
| Intensity | Ex vs. Sep | pFDR = 0.3307 |
|  | Inc vs. Com | pFDR = 0.3307 |
|  | Con_Ex vs. Con_Sep | pFDR = 0.7266 |
| Arousal | Ex vs. Sep | pFDR = 0.001 |
|  | Inc vs. Com | pFDR = 0.48 |
|  | Con_Ex vs. Con_Sep | pFDR = 0.9308 |
| Pain | Ex vs. Sep | pFDR = 0.0001 |
|  | Inc vs. Com | pFDR = 0.48 |
|  | Con_Ex vs. Con_Sep | pFDR = 0.3307 |

**Supporting Table S2. Core systems for PSSE, PSSS, PSSEvS and PSSEaS**

| **Lobe** | **Gyrus** | **Left and Right Hemisphere** | **lh.MNI(X,Y,Z)** | **voxel number** | | | | **rh.MNI(X,Y,Z)** | **voxel number** | | | |
| --- | --- | --- | --- | --- | --- | --- | --- | --- | --- | --- | --- | --- |
|  |  |  |  | **PSSE** | **PSSS** | **PSSEvS** | **PSSEaS** |  | **PSSE** | **PSSS** | **PSSEvS** | **PSSEaS** |
| **Frontal Lobe** | SFG, Superior Frontal Gyrus | SFG_L(R)_7_1 | -5 ,15, 54 | 18 | 0 | 3 | 149 | 7, 16, 54 | 2 | 0 | 5 | 78 |
|  |  | SFG_L(R)_7_2 | -18, 24, 53 | 58 | 2 | 7 | 75 | 22, 26, 51 | 64 | 0 | 0 | 173 |
|  |  | SFG_L(R)_7_3 | -11, 49, 40 | 84 | 0 | 134 | 0 | 13, 48, 40 | 91 | 6 | 85 | 79 |
|  |  | SFG_L(R)_7_4 | -18, -1, 65 | 6 | 0 | 11 | 13 | 20, 4, 64 | 0 | 0 | 0 | 30 |
|  |  | SFG_L(R)_7_5 | -6, -5, 58 | 41 | 102 | 0 | 243 | 7, -4, 60 | 8 | 99 | 0 | 55 |
|  |  | SFG_L(R)_7_6 | -5, 36, 38 | 259 | 28 | 258 | 47 | 6, 38, 35 | 208 | 68 | 262 | 57 |
|  |  | SFG_L(R)_7_7 | -8, 56, 15 | 109 | 1 | 308 | 4 | 8, 58, 13 | 14 | 3 | 47 | 3 |
|  | MFG, Middle Frontal Gyrus | MFG_L(R)_7_1 | -27, 43, 31 | 29 | 2 | 4 | 28 | 30, 37, 36 | 46 | 0 | 0 | 78 |
|  |  | MFG_L(R)_7_2 | -42, 13, 36 | 31 | 11 | 49 | 22 | 42, 11, 39 | 22 | 19 | 0 | 7 |
|  |  | MFG_L(R)_7_3 | -28, 56, 12 | 0 | 8 | 10 | 0 | 28, 55, 17 | 0 | 0 | 1 | 130 |
|  |  | MFG_L(R)_7_4 | -41, 41, 16 | 6 | 0 | 0 | 103 | 42, 44, 14 | 0 | 0 | 0 | 69 |
|  |  | MFG_L(R)_7_5 | -33, 23, 45 | 342 | 6 | 302 | 162 | 42, 27, 39 | 7 | 13 | 0 | 47 |
|  |  | MFG_L(R)_7_6 | -32, 4, 55 | 30 | 10 | 36 | 118 | 34, 8, 54 | 5 | 0 | 0 | 98 |
|  |  | MFG_L(R)_7_7 | -26, 60, -6 | 2 | 83 | 0 | 14 | 25, 61, -4 | 76 | 3 | 3 | 104 |
|  | IFG, Inferior Frontal Gyrus | IFG_L(R)_6_1 | -46, 13, 24 | 14 | 79 | 49 | 25 | 45, 16, 25 | 2 | 0 | 0 | 178 |
|  |  | IFG_L(R)_6_2 | -47, 32, 14 | 0 | 28 | 0 | 153 | 48, 35, 13 | 0 | 0 | 0 | 40 |
|  |  | IFG_L(R)_6_3 | -53, 23, 11 | 0 | 15 | 1 | 1 | 54, 24, 12 | 0 | 0 | 0 | 10 |
|  |  | IFG_L(R)_6_4 | -49, 36, -3 | 0 | 37 | 0 | 116 | 51, 36, -1 | 0 | 0 | 0 | 2 |
|  |  | IFG_L(R)_6_5 | -39, 23, 4 | 0 | 0 | 0 | 4 | 42, 22, 3 | 5 | 2 | 7 | 111 |
|  |  | IFG_L(R)_6_6 | -52, 13, 6 | 0 | 0 | 0 | 0 | 54, 14, 11 | 2 | 0 | 2 | 81 |
|  | OrG, Orbital Gyrus | OrG_L(R)_6_1 | -7, 54, -7 | 0 | 0 | 0 | 39 | 6, 47, -7 | 0 | 9 | 0 | 123 |
|  |  | OrG_L(R)_6_2 | -36, 33, -16 | 32 | 0 | 0 | 10 | 40, 39, -14 | 22 | 0 | 0 | 69 |
|  |  | OrG_L(R)_6_3 | -23, 38, -18 | 33 | 0 | 12 | 34 | 23, 36, -18 | 13 | 0 | 6 | 32 |
|  |  | OrG_L(R)_6_4 | -6, 52, -19 | 5 | 0 | 0 | 99 | 6, 57, -16 | 9 | 27 | 0 | 56 |
|  |  | OrG_L(R)_6_5 | -10, 18, -19 | 12 | 20 | 33 | 6 | 9, 20, -19 | 21 | 0 | 9 | 16 |
|  |  | OrG_L(R)_6_6 | -41, 32, -9 | 26 | 0 | 0 | 61 | 42, 31, -9 | 34 | 0 | 0 | 241 |
|  | PrG, Precentral Gyrus | PrG_L(R)_6_1 | -49, -8, 39 | 18 | 2 | 75 | 145 | 55, -2, 33 | 3 | 0 | 64 | 0 |
|  |  | PrG_L(R)_6_2 | -32, -9, 58 | 17 | 6 | 0 | 284 | 33, -7, 57 | 56 | 23 | 37 | 75 |
|  |  | PrG_L(R)_6_3 | -26, -25, 63 | 4 | 0 | 0 | 1 | 34, -19, 59 | 236 | 0 | 0 | 194 |
|  |  | PrG_L(R)_6_4 | -13, -20, 73 | 0 | 0 | 0 | 12 | 15, -22, 71 | 6 | 15 | 0 | 35 |
|  |  | PrG_L(R)_6_5 | -52, 0, 8 | 4 | 0 | 0 | 99 | 54, 4, 9 | 44 | 16 | 53 | 4 |
|  |  | PrG_L(R)_6_6 | -49, 5, 30 | 0 | 16 | 8 | 46 | 51, 7, 30 | 68 | 1 | 12 | 285 |
|  | PCL, Paracentral Lobule | PCL_L(R)_2_1 | -8, -38, 58 | 0 | 31 | 21 | 18 | 10, -34, 54 | 0 | 147 | 15 | 0 |
|  |  | PCL_L(R)_2_2 | -4, -23, 61 | 0 | 0 | 0 | 6 | 5, -21, 61 | 0 | 3 | 0 | 0 |
| **Temporal Lobe** | STG, Superior Temporal Gyrus | STG_L(R)_6_1 | -32, 14, -34 | 0 | 0 | 3 | 13 | 31, 15, -34 | 0 | 2 | 0 | 20 |
|  |  | STG_L(R)_6_2 | -54, -32, 12 | 4 | 4 | 17 | 3 | 54, -24, 11 | 48 | 0 | 60 | 60 |
|  |  | STG_L(R)_6_3 | -50, -11, 1 | 18 | 0 | 73 | 16 | 51, -4, -1 | 96 | 195 | 215 | 32 |
|  |  | STG_L(R)_6_4 | -62, -33, 7 | 69 | 276 | 196 | 305 | 66, -20, 6 | 43 | 51 | 117 | 61 |
|  |  | STG_L(R)_6_5 | -45, 11, -20 | 37 | 15 | 8 | 79 | 47, 12, -20 | 19 | 36 | 5 | 313 |
|  |  | STG_L(R)_6_6 | -55, -3, -10 | 57 | 225 | 282 | 183 | 56, -12, -5 | 32 | 203 | 99 | 224 |
|  | MTG, Middle Temporal Gyrus | MTG_L(R)_4_1 | -65, -30, -12 | 3 | 24 | 23 | 148 | 65, -29, -13 | 2 | 0 | 0 | 6 |
|  |  | MTG_L(R)_4_2 | -53, 2, -30 | 12 | 61 | 6 | 45 | 51, 6, -32 | 61 | 20 | 0 | 140 |
|  |  | MTG_L(R)_4_3 | -59, -58, 4 | 2 | 114 | 102 | 282 | 60, -53, 3 | 13 | 191 | 240 | 119 |
|  |  | MTG_L(R)_4_4 | -58, -20, -9 | 14 | 441 | 159 | 737 | 58, -16, -10 | 18 | 99 | 30 | 476 |
|  | ITG, Inferior Temporal Gyrus | ITG_L(R)_7_1 | -45, -26, -27 | 0 | 0 | 0 | 0 | 46, -14, -33 | 0 | 55 | 16 | 73 |
|  |  | ITG_L(R)_7_2 | -51, -57, -15 | 2 | 24 | 26 | 24 | 53, -52, -18 | 24 | 65 | 3 | 115 |
|  |  | ITG_L(R)_7_3 | -43, -2, -41 | 23 | 10 | 4 | 51 | 40, 0, -43 | 2 | 28 | 0 | 5 |
|  |  | ITG_L(R)_7_4 | -56, -16, -28 | 1 | 14 | 0 | 9 | 55, -11, -32 | 0 | 8 | 0 | 100 |
|  |  | ITG_L(R)_7_5 | -55, -60, -6 | 1 | 105 | 150 | 15 | 54, -57, -8 | 24 | 323 | 112 | 204 |
|  |  | ITG_L(R)_7_6 | -59, -42, -16 | 0 | 9 | 0 | 69 | 61, -40, -17 | 17 | 20 | 0 | 171 |
|  |  | ITG_L(R)_7_7 | -55, -31, -27 | 4 | 4 | 0 | 0 | 54, -31, -26 | 0 | 8 | 1 | 135 |
|  | FuG, Fusiform Gyrus | FuG_L(R)_3_1 | -33, -16, -32 | 194 | 15 | 95 | 271 | 33, -15, -34 | 199 | 27 | 125 | 237 |
|  |  | FuG_L(R)_3_2 | -31, -64, -14 | 519 | 605 | 726 | 907 | 31, -62, -14 | 494 | 388 | 673 | 510 |
|  |  | FuG_L(R)_3_3 | -42, -51, -17 | 195 | 361 | 311 | 381 | 43, -49, -19 | 33 | 84 | 63 | 91 |
|  | PhG, Parahippocampal Gyrus | PhG_L(R)_6_1 | -27, -7, -34 | 0 | 0 | 0 | 58 | 28, -8, -33 | 0 | 4 | 0 | 16 |
|  |  | PhG_L(R)_6_2 | -25, -25, -26 | 11 | 0 | 0 | 2 | 26, -23, -27 | 10 | 10 | 12 | 10 |
|  |  | PhG_L(R)_6_3 | -28, -32, -18 | 80 | 3 | 3 | 108 | 30, -30, -18 | 76 | 0 | 22 | 60 |
|  |  | PhG_L(R)_6_4 | -19, -12, -30 | 0 | 0 | 0 | 1 | 19, -10, -30 | 0 | 1 | 1 | 4 |
|  |  | PhG_L(R)_6_5 | -23, 2, -32 | 0 | 0 | 0 | 2 | 22, 1, -36 | 0 | 0 | 11 | 14 |
|  |  | PhG_L(R)_6_6 | -17, -39, -10 | 53 | 0 | 16 | 50 | 19, -36, -11 | 220 | 9 | 112 | 168 |
|  | pSTS, posterior Superior Temporal Sulcus | pSTS_L(R)_2_1 | -54, -40, 4 | 6 | 203 | 57 | 298 | 53, -37, 3 | 16 | 8 | 4 | 130 |
|  |  | pSTS_L(R)_2_2 | -52, -50, 11 | 0 | 0 | 4 | 135 | 57, -40, 12 | 154 | 0 | 121 | 100 |
| **Parietal Lobe** | SPL, Superior Parietal Lobule | SPL_L(R)_5_1 | -16, -60, 63 | 175 | 0 | 48 | 74 | 19, -57, 65 | 145 | 3 | 45 | 170 |
|  |  | SPL_L(R)_5_2 | -15, -71, 52 | 311 | 1 | 206 | 188 | 19, -69, 54 | 304 | 2 | 254 | 156 |
|  |  | SPL_L(R)_5_3 | -33, -47, 50 | 0 | 124 | 295 | 55 | 35, -42, 54 | 50 | 102 | 0 | 472 |
|  |  | SPL_L(R)_5_4 | -22, -47, 65 | 0 | 1 | 19 | 11 | 23, -43, 67 | 6 | 13 | 0 | 46 |
|  |  | SPL_L(R)_5_5 | -27, -59, 54 | 0 | 0 | 21 | 3 | 31, -54, 53 | 259 | 115 | 0 | 504 |
|  | IPL, Inferior Parietal Lobule | IPL_L(R)_6_1 | -34, -80, 29 | 590 | 95 | 166 | 793 | 45, -71, 20 | 484 | 96 | 396 | 505 |
|  |  | IPL_L(R)_6_2 | -38, -61, 46 | 38 | 34 | 69 | 152 | 39, -65, 44 | 394 | 11 | 43 | 551 |
|  |  | IPL_L(R)_6_3 | -51, -33, 42 | 237 | 188 | 381 | 274 | 47, -35, 45 | 306 | 115 | 3 | 1083 |
|  |  | IPL_L(R)_6_4 | -56, -49, 38 | 0 | 24 | 15 | 418 | 57, -44, 38 | 115 | 5 | 15 | 291 |
|  |  | IPL_L(R)_6_5 | -47, -65, 26 | 28 | 11 | 125 | 125 | 53, -54, 25 | 382 | 16 | 318 | 411 |
|  |  | IPL_L(R)_6_6 | -53, -31, 23 | 259 | 265 | 548 | 1 | 55, -26, 26 | 67 | 0 | 0 | 259 |
|  | Pcun, Precuneus | PCun_L(R)_4_1 | -5, -63, 51 | 525 | 0 | 19 | 459 | 6, -65, 51 | 386 | 0 | 91 | 190 |
|  |  | PCun_L(R)_4_2 | -8, -47, 57 | 259 | 26 | 52 | 295 | 7, -47, 58 | 295 | 66 | 80 | 199 |
|  |  | PCun_L(R)_4_3 | -12, -67, 25 | 317 | 48 | 18 | 374 | 16, -64, 25 | 597 | 10 | 194 | 571 |
|  |  | PCun_L(R)_4_4 | -6, -55, 34 | 143 | 111 | 109 | 323 | 6, -54, 35 | 230 | 95 | 103 | 437 |
|  | PoG, Postcentral Gyrus | PoG_L(R)_4_1 | -50, -16, 43 | 0 | 25 | 108 | 9 | 50, -14, 44 | 136 | 0 | 14 | 98 |
|  |  | PoG_L(R)_4_2 | -56, -14, 16 | 80 | 0 | 112 | 8 | 56, -10, 15 | 0 | 0 | 10 | 0 |
|  |  | PoG_L(R)_4_3 | -46, -30, 50 | 11 | 75 | 174 | 20 | 48, -24, 48 | 30 | 0 | 0 | 265 |
|  |  | PoG_L(R)_4_4 | -21, -35, 68 | 0 | 0 | 3 | 26 | 20, -33, 69 | 41 | 81 | 0 | 28 |
| **Insular Lobe** | INS, Insular Gyrus | INS_L(R)_6_1 | -36, -20, 10 | 0 | 0 | 0 | 8 | 37, -18, 8 | 0 | 0 | 0 | 1 |
|  |  | INS_L(R)_6_2 | -32, 14, -13 | 22 | 0 | 0 | 11 | 33, 14, -13 | 7 | 0 | 0 | 60 |
|  |  | INS_L(R)_6_3 | -34, 18, 1 | 4 | 0 | 0 | 16 | 36, 18, 1 | 10 | 0 | 0 | 171 |
|  |  | INS_L(R)_6_4 | -38, -4, -9 | 1 | 0 | 1 | 40 | 39, -2, -9 | 0 | 0 | 0 | 12 |
|  |  | INS_L(R)_6_5 | -38, -8, 8 | 0 | 0 | 0 | 25 | 39, -7, 8 | 1 | 0 | 7 | 0 |
|  |  | INS_L(R)_6_6 | -38, 5, 5 | 7 | 0 | 0 | 5 | 38, 5, 5 | 6 | 0 | 15 | 0 |
| **Limbic Lobe** | CG, Cingulate Gyrus | CG_L(R)_7_1 | -4, -39, 31 | 58 | 100 | 146 | 8 | 4, -37, 32 | 58 | 60 | 93 | 20 |
|  |  | CG_L(R)_7_2 | -3, 8, 25 | 0 | 5 | 4 | 1 | 5, 22, 12 | 0 | 0 | 0 | 21 |
|  |  | CG_L(R)_7_3 | -6, 34, 21 | 0 | 0 | 4 | 0 | 5, 28, 27 | 0 | 0 | 1 | 14 |
|  |  | CG_L(R)_7_4 | -8, -47, 10 | 0 | 177 | 81 | 217 | 9, -44, 11 | 54 | 40 | 23 | 131 |
|  |  | CG_L(R)_7_5 | -5, 7, 37 | 0 | 0 | 0 | 0 | 4, 6, 38 | 0 | 0 | 3 | 0 |
|  |  | CG_L(R)_7_6 | -7, -23, 41 | 17 | 17 | 95 | 7 | 6, -20, 40 | 21 | 9 | 80 | 9 |
|  |  | CG_L(R)_7_7 | -4, 39, -2 | 0 | 7 | 1 | 144 | 5, 41, 6 | 0 | 28 | 0 | 121 |
| **Occipital Lobe** | MVOcC*,* MedioVentral Occipital Cortex | MVOcC _L(R)_5_1 | -11, -82, -11 | 0 | 7 | 1 | 144 | 10, -85, -9 | 0 | 28 | 0 | 121 |
|  |  | MVOcC _L(R)_5_2 | -5, -81, 10 | 0 | 7 | 1 | 144 | 7, -76, 11 | 0 | 28 | 0 | 121 |
|  |  | MVOcC _L(R)_5_3 | -6, -94, 1 | 0 | 7 | 1 | 144 | 8, -90, 12 | 0 | 28 | 0 | 121 |
|  |  | MVOcC _L(R)_5_4 | -17, -60, -6 | 0 | 7 | 1 | 144 | 18, -60, -7 | 0 | 28 | 0 | 121 |
|  |  | MVOcC _L(R)_5_5 | -13, -68, 12 | 0 | 7 | 1 | 144 | 15, -63, 12 | 0 | 28 | 0 | 121 |
|  | LOcC, lateral Occipital Cortex | LOcC_L(R)_4_1 | -31, -89, 11 | 295 | 126 | 143 | 393 | 34, -86, 11 | 528 | 3 | 389 | 406 |
|  |  | LOcC _L(R)_4_2 | -46, -74, 3 | 295 | 126 | 143 | 393 | 48, -70, -1 | 528 | 3 | 389 | 406 |
|  |  | LOcC _L(R)_4_3 | -18, -99, 2 | 295 | 126 | 143 | 393 | 22, -97, 4 | 528 | 3 | 389 | 406 |
|  |  | LOcC_L(R)_4_4 | -30, -88, -12 | 309 | 1230 | 1111 | 719 | 32, -85, -12 | 139 | 494 | 591 | 158 |
|  |  | LOcC _L(R)_2_1 | -11, -88, 31 | 309 | 1230 | 1111 | 719 | 16, -85, 34 | 139 | 494 | 591 | 158 |
|  |  | LOcC _L(R)_2_2 | -22, -77, 36 | 309 | 1230 | 1111 | 719 | 29, -75, 36 | 139 | 494 | 591 | 158 |
| **Subcortical Nuclei** | Amyg, Amygdala | Amyg_L(R)_2_1 | -19, -2, -20 | 10 | 0 | 0 | 12 | 19, -2, -19 | 0 | 3 | 0 | 77 |
|  |  | Amyg_L(R)_2_2 | -27, -4, -20 | 7 | 0 | 2 | 59 | 28, -3, -20 | 13 | 1 | 10 | 63 |
|  | Hipp, Hippocampus | Hipp_L(R)_2_1 | -22, -14, -19 | 31 | 26 | 6 | 221 | 22, -12, -20 | 11 | 17 | 13 | 228 |
|  |  | Hipp_L(R)_2_2 | -28, -30, -10 | 60 | 56 | 24 | 261 | 29, -27, -10 | 78 | 6 | 39 | 96 |
|  | BG, Basal Ganglia | BG_L(R)_6_1 | -12, 14, 0 | 0 | 0 | 3 | 49 | 15, 14, -2 | 0 | 0 | 0 | 39 |
|  |  | BG_L(R)_6_2 | -22, -2, 4 | 0 | 0 | 0 | 2 | 22, -2, 3 | 40 | 0 | 0 | 34 |
|  |  | BG_L(R)_6_3 | -17, 3, -9 | 0 | 9 | 5 | 0 | 15, 8, -9 | 0 | 1 | 8 | 29 |
|  |  | BG_L(R)_6_4 | -23, 7, -4 | 0 | 0 | 0 | 11 | 22, 8, -1 | 0 | 0 | 0 | 0 |
|  |  | BG_L(R)_6_5 | -14, 2, 16 | 0 | 0 | 0 | 10 | 14, 5, 14 | 1 | 0 | 0 | 111 |
|  |  | BG_L(R)_6_6 | -28, -5, 2 | 0 | 0 | 0 | 0 | 29, -3, 1 | 3 | 0 | 0 | 0 |
|  | Tha, Thalamus | Tha_L(R)_8_1 | -7, -12, 5 | 0 | 0 | 0 | 18 | 7, -11, 6 | 70 | 6 | 43 | 29 |
|  |  | Tha_L(R)_8_2 | -18, -13, 3 | 27 | 0 | 15 | 50 | 12, -14, 1 | 26 | 2 | 24 | 11 |
|  |  | Tha_L(R)_8_3 | -18, -23, 4 | 75 | 9 | 105 | 28 | 18, -22, 3 | 25 | 5 | 62 | 5 |
|  |  | Tha_L(R)_8_4 | -7, -14, 7 | 0 | 26 | 13 | 24 | 3, -13, 5 | 29 | 0 | 24 | 51 |
|  |  | Tha_L(R)_8_5 | -16, -24, 6 | 67 | 21 | 85 | 130 | 15, -25, 6 | 98 | 13 | 83 | 79 |
|  |  | Tha_L(R)_8_6 | -15, -28, 4 | 13 | 45 | 20 | 103 | 13, -27, 8 | 85 | 11 | 94 | 42 |
|  |  | Tha_L(R)_8_7 | -12, -22, 13 | 4 | 5 | 2 | 47 | 10, -14, 14 | 52 | 0 | 22 | 77 |
|  |  | Tha_L(R)_8_8 | -11, -14, 2 | 95 | 0 | 54 | 86 | 13, -16, 7 | 115 | 15 | 98 | 39 |

**Supporting Table S3. Core systems for PSSEvS, FEPain, and NSPain**

| **Lobe** | **Gyrus** | **voxel number** | | |
| --- | --- | --- | --- | --- |
|  |  | **PSSEaS** | **FEPain** | **NSPain** |
| Frontal Lobe | SFG, Superior Frontal Gyrus | 0 | 0 | 3 |
|  | MFG, Middle Frontal Gyrus | 0 | 0 | 0 |
|  | IFG, Inferior Frontal Gyrus | 25 | 4 | 36 |
|  | OrG, Orbital Gyrus | 53 | 0 | 22 |
|  | PrG, Precentral Gyrus | 85 | 3 | 38 |
|  | PCL, Paracentral Lobule | 0 | 0 | 0 |
| Temporal Lobe | STG, Superior Temporal Gyrus | 19 | 0 | 7 |
|  | MTG, Middle Temporal Gyrus | 191 | 7 | 38 |
|  | ITG, Inferior Temporal Gyrus | 36 | 1 | 0 |
|  | FuG, Fusiform Gyrus | 20 | 0 | 0 |
|  | PhG, Parahippocampal Gyrus | 3 | 0 | 0 |
|  | pSTS, posterior Superior Temporal Sulcus | 59 | 29 | 0 |
| Parietal Lobe | SPL, Superior Parietal Lobule | 0 | 0 | 0 |
|  | IPL, Inferior Parietal Lobule | 297 | 39 | 136 |
|  | PCun, Precuneus | 36 | 0 | 0 |
|  | PoG, Postcentral Gyrus | 8 | 0 | 21 |
| Insular Lobe | INS, Insular Gyrus | 36 | 86 | 120 |
| Limbic Lobe | CG, Cingulate Gyrus | 7 | 0 | 0 |
| Occipital Lobe | MVOcC, MedioVentral Occipital Cortex | 0 | 0 | 0 |
|  | LOcC, lateral Occipital Cortex | 132 | 0 | 0 |
| Subcortical Nuclei | Amyg, Amygdala | 35 | 0 | 0 |
|  | Hipp, Hippocampus | 16 | 0 | 0 |
|  | BG, Basal Ganglia | 0 | 0 | 0 |
|  | Tha, Thalamus | 0 | 0 | 0 |

**Supporting Table S4. Similarity of neural representations of different ROIs between decoders**

| **Region** | **Decoders weight patterns similarity** | | |
| --- | --- | --- | --- |
|  | **PSSEaS and FEPain** | **PSSEaS and NSPain** | **PSSEaS and REPain** |
| Left anterior insula | r = 0.2195 pFDR < 0.001 | r = 0.3365 pFDR < 0.001 | r = -0.3436 pFDR < 0.001 |
| Right anterior insula | r = -0.4416 pFDR = 0.0093 | r = -0.5682 pFDR < 0.001 | r = -0.2277 pFDR = 0.1752 |
| Left cerebellum | r = -0.2892 pFDR < 0.001 | r = -0.2663 pFDR < 0.001 | r = -0.0828 pFDR = 0.0880 |
| Right cerebellum | r = 0.5909 pFDR < 0.001 | r = 0.4025 pFDR < 0.001 | r = 0.1285 p = 0.0275 |
| Precuneus | r = 0.3482 pFDR < 0.001 | r = -0.5405 pFDR < 0.001 | r = -0.6284 pFDR < 0.001 |
| Periaqueductal Gray | r = -0.1693 pFDR = 0.0069 | r = -0.0801 pFDR = 0.1824 | r = -0.5326 pFDR < 0.001 |
| Rectus | r = -0.1876 pFDR = 0.0935 | r = 0.0933 pFDR = 0.4072 | r = 0.5390 pFDR < 0.001 |
| Dorsomedial Prefrontal Cortex | r = -0.0134 pFDR = 0.7837 | r = 0.0478 pFDR = 0.3282 | r = -0.1332 pFDR = 0.0189 |

**Supporting Table S5. Forced-choice classification performance across own datasets.**

| Forced-choice discrimination test | | Discrimination | Effect size | | Binomial test |
| --- | --- | --- | --- | --- | --- |
|  |  | Accuracy | AUC | d_a_ | P-value |
| Exclusion vs. Inclusion in discovery cohort | PSSEaS | 100%± 0.0% (SE) | 1 | 4.82 | pFDR < 0.001 |
|  | PSSEvS | 89% ± 2.7% (SE) | 0.85 | 1.11 | pFDR < 0.001 |
|  | FEPain | 65% ± 4.2% (SE) | 0.67 | 0.51 | pFDR = 0.0019 |
|  | NSPain | 63% ± 4.2% (SE) | 0.62 | 0.4 | pFDR = 0.0051 |
|  | REPain | 56% ± 4.4% (SE) | 0.55 | 0.19 | pFDR = 0.1881 |
|  | HEPain | 66% ± 4.2% (SE) | 0.7 | 0.64 | pFDR < 0.001 |
|  | SIIPS1 | 62% ± 4.3% (SE) | 0.66 | 0.55 | pFDR = 0.0074 |
| Exclusion vs. Inclusion in replication cohort | PSSEaS | 99% ± 1.4% (SE) | 1 | 3.63 | pFDR < 0.001 |
|  | PSSEvS | 70% ± 5.5% (SE) | 0.72 | 0.59 | pFDR = 0.0026 |
|  | FEPain | 64% ± 5.7% (SE) | 0.66 | 0.59 | pFDR = 0.0262 |
|  | NSPain | 67% ± 5.6% (SE) | 0.7 | 0.68 | pFDR = 0.0078 |
|  | REPain | 61% ± 5.8% (SE) | 0.6 | 0.37 | pFDR = 0.0722 |
|  | HEPain | 70% ± 5.5% (SE) | 0.74 | 0.75 | pFDR = 0.0026 |
|  | SIIPS1 | 67% ± 5.6% (SE) | 0.69 | 0.71 | pFDR = 0.0078 |
| Separation  vs. Company  in discovery cohort | PSSEaS | 100% ± 0.0% (SE) | 1 | 5.33 | pFDR < 0.001 |
|  | PSSEvS | 100% ± 0.0% (SE) | 1 | 3.15 | pFDR < 0.001 |
|  | FEPain | 69% ± 4.0% (SE) | 0.71 | 0.77 | pFDR < 0.001 |
|  | NSPain | 71% ± 4.0% (SE) | 0.77 | 1.02 | pFDR < 0.001 |
|  | REPain | 65% ± 4.2% (SE) | 0.68 | 0.57 | pFDR = 0.0015 |
|  | HEPain | 56% ± 4.4% (SE) | 0.57 | 0.26 | pFDR = 0.2195 |
|  | SIIPS1 | 55% ± 4.4% (SE) | 0.52 | 0.03 | pFDR = 0.2541 |
| Separation  vs. Company in replication cohort | PSSEaS | 99% ± 1.4% (SE) | 1 | 3.42 | pFDR < 0.001 |
|  | PSSEvS | 86% ± 4.2% (SE) | 0.94 | 2.02 | pFDR < 0.001 |
|  | FEPain | 66% ± 5.7% (SE) | 0.66 | 0.54 | pFDR = 0.0161 |
|  | NSPain | 67% ± 5.6% (SE) | 0.69 | 0.66 | pFDR = 0.013 |
|  | REPain | 61% ± 5.8% (SE) | 0.61 | 0.39 | pFDR = 0.0843 |
|  | HEPain | 66% ± 5.7% (SE) | 0.68 | 0.66 | pFDR = 0.0161 |
|  | SIIPS1 | 60% ± 5.9% (SE) | 0.58 | 0.29 | pFDR = 0.1196 |

**Note.** Forced-choice discrimination, the decision threshold (for the difference between pairs) is 0, The discrimination accuracy is expressed as: accuracy ± standard error (SE). AUC: Area under the Receiver Operating Characteristic curve, a threshold-independent measure of performance; chance is 0.5. d_a_: Discriminability, a measure.

**Supporting Table S6. Forced-choice classification performance across other datasets.**

| Forced-choice discrimination test | | Discrimination | Effect size | | Binomial test |
| --- | --- | --- | --- | --- | --- |
|  |  | Accuracy | AUC | da | P-value |
| Validation cohort1: FE physical vicarious pain | PSSE | 53% ± 2.3% (SE) | 0.5 | 0.02 | pFDR = 0.2918 |
|  | PSSS | 56% ± 2.3% (SE) | 0.57 | 0.24 | pFDR = 0.0102 |
|  | PSSEaS | 55% ± 2.3% (SE) | 0.54 | 0.12 | pFDR = 0.0232 |
|  | PSSEvS | 57% ± 2.3% (SE) | 0.58 | 0.26 | pFDR = 0.0057 |
|  | FEPain | 100% ± 0.0% (SE) | 1 | 4.39 | pFDR < 0.001 |
|  | NSPain | 66% ± 2.2% (SE) | 0.71 | 0.75 | pFDR < 0.001 |
| Validation cohort2: NS physical vicarious pain | PSSE | 57% ± 2.3% (SE) | 0.57 | 0.22 | pFDR = 0.0025 |
|  | PSSS | 65% ± 2.2% (SE) | 0.69 | 0.67 | pFDR = 0.0025 |
|  | PSSEaS | 57% ± 2.3% (SE) | 0.58 | 0.28 | pFDR = 0.0193 |
|  | PSSEvS | 66% ± 2.2% (SE) | 0.71 | 0.74 | pFDR < 0.001 |
|  | FEPain | 72% ± 2.1% (SE) | 0.79 | 1.1 | pFDR < 0.001 |
|  | NSPain | 100% ± 0.2% (SE) | 1 | 4.01 | pFDR < 0.001 |
| Generalization cohort 1: Episodic physical pain | PSSE | 60% ± 3.9% (SE) | 0.59 | 0.21 | pFDR = 0.0245 |
|  | PSSS | 62% ± 3.9% (SE) | 0.63 | 0.33 | pFDR = 0.0139 |
|  | PSSEaS | 59% ± 4.0% (SE) | 0.56 | 0.21 | pFDR = 0.0351 |
|  | PSSEvS | 64% ± 3.9% (SE) | 0.63 | 0.4 | pFDR = 0.0054 |
|  | FEPain | 58% ± 4.0% (SE) | 0.58 | 0.2 | pFDR = 0.0635 |
|  | NSPain | 60% ± 4.0% (SE) | 0.61 | 0.35 | pFDR = 0.0287 |
| Generalization cohort 2: Subjective disgust (n = 78) | PSSE | 59% ± 2.5% (SE) | 0.55 | 0.15 | pFDR = 0.9612 |
|  | PSSS | 63% ± 2.5% (SE) | 0.52 | 0.07 | pFDR = 0.8673 |
|  | PSSEaS | 62% ± 2.5% (SE) | 0.57 | 0.21 | pFDR = 0.9612 |
|  | PSSEvS | 60% ± 2.5% (SE) | 0.52 | 0.07 | pFDR = 0.9612 |
|  | FEPain | 64% ± 2.4% (SE) | 0.6 | 0.35 | pFDR = 0.8673 |
|  | NSPain | 63% ± 2.5% (SE) | 0.61 | 0.36 | pFDR = 0.8673 |

**Note.** For two-choice (forced-choice) discrimination, the decision threshold (for the difference between pairs) is 0, The discrimination accuracy is expressed as: accuracy ± standard error (SE). AUC: Area under the Receiver Operating Characteristic curve, a threshold-independent measure of performance; chance is 0.5. d_a_: Discriminability, a measure.
